# Heat Shock Protein 60 Antibodies Are Associated With a Risk Factor for Cardiovascular Disease in Bedridden Elderly Patients

**DOI:** 10.3389/fmolb.2020.00103

**Published:** 2020-06-12

**Authors:** Jonas Bernardes de Lima Filho, Letícia Freire, Eliana Aguiar Petri Nahas, Fábio Lera Orsatti, Claudio Lera Orsatti

**Affiliations:** ^1^Department of Health Science, Oeste Paulista University – UNOESTE, Jau, Brazil; ^2^Department of Gynecology and Obstetrics, Botucatu Medical School, Paulista State University (UNESP), Botucatu, Brazil; ^3^Department of Sport Sciences, Health Science Institute, Federal University of Triangulo Mineiro (UFTM), Uberaba, Brazil

**Keywords:** bedridden elderly, HSP60, anti-HSP60 antibody, risk factors, cardiovascular disease

## Abstract

Frailty, in elderly people, represents multiple deficiencies in different organs and is characterized by decreased physiological reserves and greater vulnerability to stressors. Bedridden elderly, with cardiovascular disease (CVD), have a worse prognosis than non-bedridden patients. Heat-shock proteins (HSPs) are molecular chaperones that under physiological conditions facilitate the transport, folding and assembly of proteins. Serum HSP 60-kDa concentrations and their antibodies are increased, in response to non-physiological conditions, suggesting the involvement of HSPs and their antibodies in the development of CVD. The aim of this work was to evaluate heat shock protein 60 and anti-HSP60 antibody levels, associated with a risk factor for cardiovascular disease, in bedridden elderly patients. Clinical, analytical and cross-sectional analyses were performed with 57 elderly (>65 years). HSP60 and anti-HSP60 plasma levels were measured by ELISA. Bivariate analysis using a linear regression model adjusted for risk factors used Framingham Score. Among the 57 elderly, with an average age of 69.89 years, 39% are bedridden; 26% with pre-existing cardiovascular disease and 44% are dyslipidemic. The relationship of risk factors in the Framingham Score was positive for the anti-HSP60 antibody (*p* = 0.042) measurement. Our data show a positive correlation among the elevation of the Framingham score and the profile of anti-HSP60 antibodies. These results suggest a greater immune activation that is associated with cardiovascular risk and bedridden fragility.

## Introduction

The progressive decrease of functional physiological ability, that occurs as consequence of the aging process, has been consistently associated with the significant reduction of life quality, cognitive function decrease, falls with bone fractures, thromboembolism and risk factors for cardiovascular disease (CVD), the last being the main cause of elderly people mortality (Di Nisio et al., 2011; Evans, 2011; Ortmann and Lattrich, 2012). CVD is a progressive disease that begins as subclinical atherosclerosis during a long asymptomatic phase (Vasan, 2006). The development of the CVD is mainly associated with three important risk factors: diabetes, systemic arterial hypertension and dyslipidemia (Topinkov, 2008). Thus, additional tools aiding the clinical assessment and to improve their ability to identify patients with risk factors, for CVD, are necessary (Akita Chun and McGee, 2004; Vasan, 2006). It has been suggested that measurable and quantifiable biological parameters (biomarkers) are useful tools to aid in the disease prognosis and to identify disease risks and metabolic processes (Vasan, 2006).

Heat shock proteins (HSPs) are expressed by homeostatic cells with molecular protective function against cell damage (Delogu, 2000; Niizeki et al., 2008; Martine and Rébé, 2019). Although HSPs can be released into the bloodstream, under stress conditions (Zhu et al., 2001; Pockley, 2002), the HSPs are responsible for deleterious effects on the arterial wall, such as endothelial cell activation, promoting atherosclerosis (Zhu et al., 2001; Rodríguez-Iturbe and Johnson, 2018). HSPs, in body fluids, are potential biomarkers detectable in tissue-damaged diseases, that activate immune-inflammatory responses (Taha et al., 2019), it is suggested that HSPs may be a potential immunological component from the atherosclerosis development (Pockley et al., 2002; Lu and Kakkar, 2010; Wick et al., 2014; Santovito and Weber, 2017; Rodríguez-Iturbe and Johnson, 2018). The HSP subfamily of HSP60 and the anti-HSP60 antibodies have been studied in patients with atherosclerosis, relating them to the disease severity and progression (Zhu et al., 2001; Damluji et al., 2015; Galović et al., 2016; Rodríguez-Iturbe and Johnson, 2018). It is possible that HSP60 is a powerful molecular biomarker that activates cells involved in atherosclerosis, including vascular epithelial, smooth muscular cells, B and T lymphocytes, which are, altogether, associated with the risk factors related to CVD beginning processes (Xu et al., 2000; Lu and Kakkar, 2010). Clinical studies regarding HSP60, antibody anti-HSP60 and risk factors for atherosclerosis are controversial, demonstrating the relevance of further studies especially focused on elderly with risk factors for CVD developments (Terry et al., 2004; Ellins et al., 2008). Anti-HSP60 is an inflammatory and risk marker for atherosclerosis, and its higher serum values were associated with increased CVD. These inflammatory biomarkers may have a prognostic role in cardiovascular disease, particularly in the early detection of asymptomatic atherosclerosis (Akita Chun and McGee, 2004; Banecka-Majkutewicz et al., 2014; Damluji et al., 2015).

The role of HSP activity as a risk factor for the CVD and specifically referent to elderly patients is still controversial. Elderly people demonstrate an apparent decrease in HSP60 and Hsp70 and that does not appear to be related to anti-heat shock protein antibody status (Rea et al., 2001). The study authors also report that further studies are required to understand the basis for the declining serum HSP60 and Hsp70 levels in aging (Rea et al., 2001). Based on this context, the aim of this study was to evaluate the HSP60 and anti HSP60 levels and their association with risk factors for CVD development in elderly people.

## Materials and Methods

### Study Design and Sample Selection

This is a clinical, analytical, and cross-sectional study. The sample size calculation was based on the Pockley et al., 2002 study, with a 95% confidence coefficient and a 5% margin of error that supports a sample size composed of at least 50 individuals (Pockley et al., 2002). A total of 57 participants were included in the study. Among these, 23 (40%) were bedridden patients and 34 (60%) move normally or with the aid of support.

In the current study, individuals of both genders, with age equal or ≥65 and no current or prior manifestation of CVD (angina, acute myocardium infarction, and stroke) were included. The exclusion criteria were: (1) drug users that affect the lipoprotein metabolism; (2) addiction to either alcohol or illicit drugs; (3) autoimmune diseases individuals; (4) aneurysmal disease; (5) peripheral arterial disease; (6) chronic kidney disease. The study was performed according to the declaration of Helsinki. Informed consent was obtained from all participants and the study was approved by the Research Ethics Committee of the Faculdades Integradas de Bauru/FIB – SP, São Paulo, Brazil (process number: 976.436-CEP). Information regarding age, smoking, alcoholism, use of hormone therapy, personal history of systemic arterial hypertension (HTN) (through the use of medications), dyslipidemia, diabetes, thyroid disease and use of any medication were collected through an interview. Family medical history of acute myocardial infarction (AMI), diabetes and CVD were also evaluated. Blood pressure was measured with a mercury manometer with the subject sitting on 3 occasions at 5-min intervals after 15 min of rest (Pockley et al., 2002).

### Anthropometry

The following information were obtained for anthropometric evaluation: weight (kg), height (meters) and body mass index (BMI = weight/height^2^). It has been used the World Health Organization standards of 2002 for patient classification, according to the BMI: <18.5 kg/m^2^ low weight, 18.5–24.9 kg/m^2^ normal weight, 25–29.9 kg/m^2^ overweight and 30 kg/m^2^ obesity (Expert Panel on Detection, Evaluation, and Treatment of High Blood Cholesterol in Adults, 2001).

### Laboratory Tests

Blood samples were collected from each subject, after 12 h of fasting. After centrifugation to remove the clot, samples underwent biochemical analysis immediately and a serum aliquot was frozen and kept at −80°C for the HSP determinations. Triglycerides (TG), total cholesterol (TC) and glucose measurements were processed by an automated analyzer, Model Vitros 950^®^, by the colorimetric dry chemistry method (Johnson & Johnson^®^, Rochester, NY, United States). The optimal values were TC < 200 mg/dl, TG < 150 mg/dl and glucose <100 mg/dL (Expert Panel on Detection, Evaluation, and Treatment of High Blood Cholesterol in Adults, 2001).

### Heat Shock Protein

The HSP60 and anti-HSP60 plasmatic concentrations were determined by immunoassay with ELISA technique, complying with the kit manufacturer recommendations (Assay Designs, Stressgen, Ann Arbor, MI, United States). The analytic sensibility for the HSP60 and anti-HSP60 determinations were 3.125 and 2.88 ng/ml, respectively. The intra and inter-assay variation coefficients were lower than 10% according to the kit descriptions. The proteins were measured in non-dilute serum and the quantifications were realized on the same plate and day to avoid inter-assay variations and perform assay standardization.

### Statistic

Results were expressed as total numbers, means, standard deviations and percentages. Bivariate analysis, using a linear regression model adjusted for risk used framingham Score, was used to evaluate the influence of HSP60 and anti-HSP60 on cardiovascular disease risk. The presence of HSP60 or anti-HSP60 values were determined as higher values than percentile 50 od sample. *p*-value <5% was considered significant.

## Results

The anthropometric and clinical characteristics of the 57 elderlies are described in Table 1. It was observed that the medium age was 69.8 and there is a prevalence from male and female individuals of 56 and 44% respectively. Among the 57 patients, 40% were bedridden and 60% could move normally or with support. From these patients, 42% are smokers, 14% are hypertensive and 22% are diabetics. Regarding the corporal composition indicators, 34 of the participants presented BMI ≥30 kg/m^2^, considered obese. Medium blood pressure was considered normal. The population showed their self-none dyslipidemia with total cholesterol medium as 167.3 mg/dL and triglycerides 129.1 mg/dL. The glucose levels were presented according to the normality with medium values of 94.3 mg/dL. The HSP60 and anti-HSP60 presented medium values of 11.5 and 52.60 ng/mL respectively (Table 1). Table 2 displays the characteristics of the 57 participating in the study.

**TABLE 1:** Clinical and anthropometric characteristics of the 57 study participants.

Variables	Mean + SD	Median	Minimum/maximum
Age, years	69.89 + 9.35	72.0	48.0-96.0
Weight, kg	82.30 + 12.98	86.0	43.0-102.0
Height, cm	165 + 0.08	160	140-180
BMI, kg/m^2^	29.95 + 5.10	30.1	13.6-43.1
SBP, mmHg	131 + 16.41	130	90-190
DBP, mmHg	76 + 12.54	75	50-90
Total cholesterol, mg/dL	167.3 + 36.73	167.3	93.0-270.0
Triglycerides, mg/dL	129.1 + 52.32	123.0	49.0-252.0
LDL, mg/dL	102.7 + 51.84	100.8	14.2-256.6
HDL, mg/dL	66.3 + 21.72	63.0	21.6-135.8
Glucose, mg/dL	94.33 + 23.40	89.0	59.0-211.0
Creatinine, mg/dL	0.89 + 0.22	0.9	0.5-1.4
HSP60, ng/mL	11.48 + 3.67	11.3	5.1-21.0
Anti-HSP60, ng/mL	52.60 + 17.60	49.8	20.4-116.8

*BMI, body mass index; SBP, systolic blood pressure; DBP, diastolic blood pressure; HDL, lipoprotein of high density; LDL, lipoprotein of low density; HSP60, Heat Shock Protein 60.*

**TABLE 2:** Baseline characteristics of the 57 study participants.

Variables	*n*	%
Gender, M/F, *n*(%)	31/26	54.38/45.62
Bedridden, *n*(%)	23	38.98
Not Caucasian, *n*(%)	4	7.02
HTN, *n*(%)	11	19.30
Cardiovascular disease, *n*(%)	15	26.32
Thyroid disease, *n*(%)	4	7.02
Dyslipidemia, *n*(%)	25	43.86
Smoking	24	42.11
Diabetes	21	36.84

*M/F, male, female; HTN, systemic arterial hypertension; n, number; %, percent.*

The relationship between the risk factors, in Framingham Score, and HSP60, anti-HSP60 and Bedridden are shown in Table 3. There was a significant association between high anti-HSP60 values (B, 0.051; DP, 0.024; Beta, 0.271; IC (95%), 0.002–0.099; *p* = 0.042) in circulating blood with cardiovascular disease. There was no significant association between HSP60 and bedridden in this bivariate analysis (Table 3).

**TABLE 3:** Relationship of risk factors in Framingham Score.

Variables	*B*	DP	Beta	IC95%	*p*
Bedridden	1.130	0.892	0.168	−0.657 to 2.917	0.210
HSP60 (ng/mL)	–0.101	0.120	–0.113	−0.341 to 0.140	0.405
ANTI-HSP60 (ng/mL)	0.051	0.024	0.271	0.002 to 0.099	0.042*

HSP60, Heat Shock Protein 60. Dependent Variable: ESCFRAM. Linear Regression through the Origin *p < 0.05.

## Discussion

There are reports that immune mechanisms are involved in the atherosclerosis pathogenesis (Wick et al., 2004; Lu and Kakkar, 2010) and it is well established that cardiovascular risk factors, such as HTN, diabetes and oxidative stress, stimulate arterial wall cells and other tissues to express and/or produce high HSP concentrations (Xu, 2002; Wick et al., 2014). Associations between antibodies, HSP, CVD risk and hypertension have been documented in several studies (Kocsis et al., 2002; Pockley et al., 2002; Zhang et al., 2008). However, biomarker studies in elderly patients are limited and mostly, non-exclusive (Bårdsen et al., 2016; Galović et al., 2016; Nakayoshi et al., 2016). In this present study, we were able to observe that anti-HSP60 plasmatic presences were associated with risk factors in the Framingham Score, one of the principal measurements for CVD in elderly patients. Our data show that at each Framingham score elevation the anti-HSP60 antibody profile increases.

The anti-HSP60 elevation on the circulation can be associated with the presence and severity of the coronary disease, atherosclerosis development, and pathologic alterations in the small brain vessels (Galović et al., 2016). *In vitro* and *in vivo* studies have demonstrated that the risk factors to atherosclerosis can cause endothelial disturbances with simultaneous expression of adhesion molecules and HSP60 on the mitochondria, cytoplasm and cells surface, where it can act as dangerous signs to cellular and humoral immune reactions (Wick et al., 2014; Juwono and Martinus, 2016). The heat influence, in elderly rats, activates the HSP60 and, as a consequence, the immune and endothelial cells, inducing macrophages to secrete a considerable amount of inflammatory cytokines (TNF-α and IL-6) and express ICAM-1, leading to inflammatory responses (Pockley, 2003). As a consequence, coronary endothelial cells can be damaged (Wick et al., 2014; Zhang et al., 2014) promoting an increased permeability of the blood vessels and reduction of superoxide dismutase (SOD) activity in cardiac tissues (Leite et al., 2004). In this manner, blood lipoproteins with large cholesterol quantity are more oxidated, then, penetrates into the artery inner layer and settles in blood vessel walls. These events cause the atherosclerosis plaque formation (Grundtman et al., 2011; Rizzo et al., 2011), which leads to the occurrence of CVD in elderly rats (Zhang et al., 2014).

A study showed a significant and independent association between higher levels of plasma HSP60 and an increase of carotid arterial rigidity. Concluding that HSP60 is a powerful activator of vascular endothelial cells and smooth muscle cells, which might trigger blood vessel alterations (Ellins et al., 2008). Thus, it proved that the age increase occurs an evident reduction in HSP60 production, which is not related to their respective antibodies, characterizing incapacity of answering to stress related to the age (Ellins et al., 2008). The analyses revealed a progressive decrease in HSP60 levels according to the age, however, uncorrelated to anti-HSP60 levels (Rea et al., 2001). This might be related to our data once our values presented low values of HSP60 (11.48 ng/mL) differently from the value of anti-HSP60 (52.6 ng/mL).

It was demonstrated anti-HSP60 antibodies are increased in the serum of patients with atherosclerosis and they are related to the disease gravity (Ellins et al., 2008; Grundtman et al., 2011). In atherosclerosis, Anti-HSP might work as a diagnose biomarker besides having a possible pathogenic role (Macario, 1995; Pockley, 2003). Endothelial cells produce large levels of HSP60 and adhesion molecules when exposed to risk factors and anti-HSPs causing lysis in the activated endothelial cells when exposed to risk factors (Schett et al., 1995). Autoimmune reactions, toward human HSP60, endothelium damage and contribute to CVD (Santovito and Weber, 2017). Additional evidence showed that immune responses may underlie the formation of atherosclerotic plaque, leading to stroke (Banecka-Majkutewicz et al., 2014). The increased concentrations of circulating anti-HSP60 suggests its involvement in diabetic macroangiopathy and correlates with parameters of endothelial cell damage (Rabczynski et al., 2012). These early changes in the atherosclerotic processes are potentially reversible as long as the parallel risk factors are removed (Pockley, 2003).

## Conclusion

In summary, the accumulation of Framingham score risk factors significantly increases the anti-HSP60 antibody profile. The progressive knowledge about the number and function of atheroma components will shed light on clinical benefits for the elderly population. The biomarker experimentation on these patients with atherosclerotic disease should be discussed in further studies.

## Data Availability Statement

All datasets generated for this study are included in the article/supplementary material.

## Ethics Statement

The studies involving human participants were reviewed and approved by Research Ethics Committee of the Faculdades Integradas de Bauru/FIB – SP, São Paulo, Brazil (process number: 976.436-CEP). The patients/participants provided their written informed consent to participate in this study.

## Author Contributions

CO, FO, and JL proceeded with the study design. LF sample selection, preceded to the collection of the anthropometric, and developed the laboratory tests. CO and FO preceded to statistical data testing procedure. CO, FO, EN, JL, and LF wrote the manuscript.

## Conflict of Interest

The authors declare that the research was conducted in the absence of any commercial or financial relationships that could be construed as a potential conflict of interest.
